# Pituitary apoplexy induced by Gonadotropin-releasing hormone agonists for treating prostate cancer-report of first Asian case

**DOI:** 10.1186/1477-7819-11-254

**Published:** 2013-10-02

**Authors:** Tsung-Yi Huang, Jih-Pin Lin, Ann-Shung Lieu, Yi-Ting Chen, Hung-Sheng Chen, Mei-Yu Jang, Jung-Tsung Shen, Wen-Jeng Wu, Shu-Pin Huang, Yung-Shun Juan

**Affiliations:** 1 Department of Urology, Kaohsiung Medical University Hospital, Kaohsiung Medical University, 100 Tz-You 1st Road, Kaohsiung, Taiwan; 2 School of Post-baccalaureate Medicine, Kaohsiung Medical University, Kaohsiung, Taiwan; 3 Division of Neurosurgery, Department of Surgery, Kaohsiung Medical University Hospital, Kaohsiung Medical University, Kaohsiung, Taiwan; 4 Department of Pathology, Kaohsiung Medical University Hospital, Kaohsiung Medical University, Kaohsiung, Taiwan; 5 Department of Obstetrics and Gynecology, Kaohsiung Medical University Hospital, Kaohsiung Medical University, Kaohsiung, Taiwan; 6 Department of Urology, Kaohsiung Municipal Hsiao-Kang Hospital, Kaohsiung, Taiwan; 7 Department of Urology, College of Medicine, Kaohsiung Medical University, Kaohsiung, Taiwan

**Keywords:** Gonadotropin-releasing hormone agonists, Pituitary apoplexy, Prostate cancer

## Abstract

We present the first Asian case of a 77-year-old man who developed pituitary apoplexy (PA) soon after gonadotropin-releasing hormone agonist (GnRHa) (leuprorelin) injection to treat prostate cancer. Headache, ophthalmoplegia, visual field deficit, nausea, and vomiting are the typical characteristics of pituitary apoplexy. Though the occurrence rate is rare, the consequence of this condition can vary from mild symptoms such as headache to life-threatening scenarios like conscious change. Magnetic resonance imaging is the best imaging modality to detect PA and sublabial trans-sphenoid pituitary tumor removal can resolve most of PA symptoms and is so far the best solution in consensus. We also review 11 previous reported cases receiving GnRHa for androgen deprivation therapy of prostate cancer, and hope to alert clinicians to use GnRHa with caution.

## Background

Androgen deprivation therapy (ADT) is the mainstream medical treatment consisting of gonadotropin-releasing hormone agonist (GnRHa) combined with antiandrogens to avoid transient flare-up in testosterone levels in treating advanced prostate cancer (PCa) [1]. Pituitary apoplexy (PA) is bleeding or impaired blood supply of the pituitary gland at the base of the brain. This usually occurs in the presence of a tumor of the pituitary. Rarely, GRHa might induce PA in those who have insidious pituitary adenoma coincidentally, although most cases have not been diagnosed previously. Since Ando et al. reported the first PA case after GnRHa treatment in 1995 [2], there have been 10 additional cases published in the literature. The most common initial clinical symptom of PA is a sudden headache, often associated with a rapidly worsening visual field defect or double vision caused by compression of nerves surrounding the gland. If there is no prompt treatment, consciousness change or death might occur [2-5]. Sudden onset headache, nausea, vomiting, and ensuing visual impairment are major characteristics that need to be recognized.

According to previous studies, PA results from sudden hemorrhage or infarction of pituitary adenoma which appears in 10% to 20% of the general population without race predilection [6-8]. Herein, we present the first Asian male who developed PA after receiving his first dose of leuprorelin. We also reviewed the previous 11 reported cases. It is hoped this review can shed light on how to approach patients who have been injected with GnRHa and consequently suffered from pituitary apoplexy.

## Case presentation

A 77-year-old with prostate cancer confirmed by transrectal ultrasound biopsy 6 months previously with Gleason score of 6 (3 + 3) was admitted. Serum prostate-specific antigen (PSA) level at detection was 31.21 ng/mL. Magnetic resonance imaging (MRI) showed prostate cancer at the right posterior aspect of the peripheral zone with extra capsular extension and right seminal vesicle invasion with the clinical stage cT3bN_0_M_0_. He suffered from severe generalized headache followed by vomiting a coffee grounds-like substance after leuprorelin (3.75 mg) subcutaneous injection and anti-androgen therapy at noon. He was admitted to the Emergency Room (ER) because of pain at midnight. He had hypertension under regular medication control, peptic and esophageal ulcer, and old transient ischemia attack, while radiotherapy was administered for prostate cancer. He denied the habits of smoking or drinking. There was no history of migraine, or any type of headache.

Esophagogastroduodenoscopy (EGD) and brain-computed tomographic (CT) scan were arranged immediately. The former revealed lacerations at the E-C junction, and the latter showed no obvious intracerebral hemorrhage. Other initial presentations at ER were as follows: GCS was E4V5M6, blood pressure was 181/91 mmHg, and heart rate was 75 beats/min. Mallory Weiss laceration was ligated under EGD, but the cause of persistent headache was still a puzzle. Ophthalmologic examination manifested left cranial nerve III palsy, ptosis and eye movement limitation, and newly onset left-side blurred vision (VOS: 0.2 ^-2^ ). Funduscopy showed cup-to-disc ratio of both eyes was 0.8/0.8. Both eye visual field defects were shown by auto perimetry. Neurologic examination revealed normal muscle tone, muscle power, and intact deep tendon reflex. Laboratory examination revealed normal serum cortisol, luteinizing hormone, prolactin, insulin like growth factor-1, and testosterone levels. However, hyponatremia (Na 123 mmol/L) and high follicle stimulating hormone level (55.3 mIU/mL; normal range 1–19 mIU/mL) were noted.

**Figure 1:**
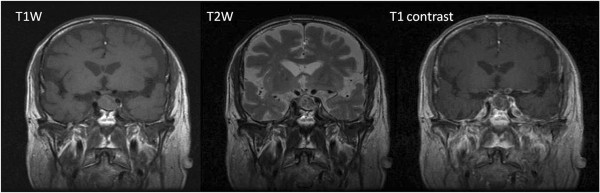
**The enlarged pituitary gland with maximal size of 1.8 cm is noted in MRI examination.** The signal characteristic of enlarged pituitary gland is non-specific, including hypointense signal on T2**-**weighted images and intermediate signal on T1-weighted images, with no water restriction on diffusion-weighted imaging. The most specific finding is non-enhancement of the pituitary gland, suggestive of infarction or so-called apoplexy.

**Figure 2:**
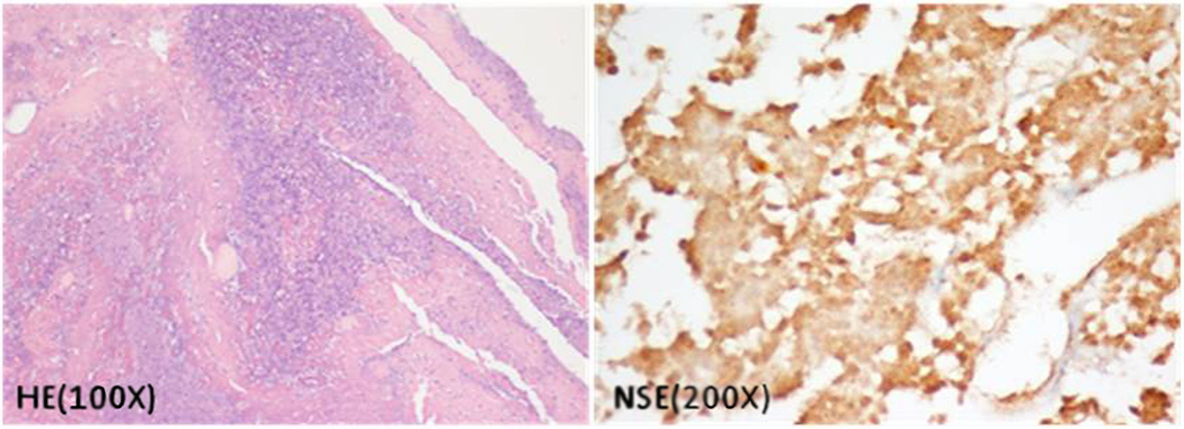
**Microscopic findings of the sections show degenerative cells with necrotic debris, fibrinoid materials, and mixed inflammatory cells.** The NSE stain only discloses the shadows of dead pituitary gland cells. The morphology is compatible with apophysial status.

Brain MRI revealed an enlarged pituitary gland (1.7 × 1.8 × 1.3 cm) with low signal intensity lesion on T1W images and slightly hyper intense on T2W images, indicating the development of pituitary gland infarction (Figure 1). Due to disease progression, on the 10th day after GnRHa injection, the patient underwent sub labial trans-sphenoid pituitary tumor removal with endoscopy under microscope. Pathological sections exhibited degenerative cells with necrotic debris, fibrinoid materials, and mixed inflammatory cells (Figure 2). Neuron Specific Enolase stain failed to demonstrate any surviving pituitary gland cells, confirming the development of PA.

Two months later, his PSA decreased to 0.46 ng/mL. Moreover, 4 months after surgery, his visual field, range of EOM, and visual acuity of both eyes had improved tremendously, and his C/D ratio had returned to 0.6/0.6. His recovery to date is almost complete.

## Conclusions

The occurrence of PA can be induced by several factors, such as head trauma, cardiac bypass surgery associated with fluctuating blood pressure, pregnancy, Sheehan syndrome, anticoagulation, pituitary function test, or as in our case, GnRHa used for treating PCa [9]. The causal relationship of GnRHa and PA is established on time relationship and consistency association. The presented case is the first Asian male suffering from PA due to GnRHa administration, illustrating no race predilection.

All PA cases reviewed except the present case were confirmed to all have pituitary adenoma before GnRHa administration. Symptoms struck most patients in 1 day immediately after first injection of GnRHa, and only two cases had PA onset later on the 9th and 10th days following treatment. Guerra has described this phenomenon as a biphasic phase, with the first peak attacking within 4 h and the second peak delayed even more than 1 week [10]. The exact mechanism of GnRHa-induced PA is still unclear. Multiple factors might contribute to the increased risk of bleeding associated with GnRHa usage. Intrinsic pituitary vasculature abnormalities, larger size of adenoma, and elevated intrasellar pressure can account for increased thickness and edematous change in capillaries, rapid tumor growth, and decrease in blood flow resulting in following ischemic change or bleeding. This does make sense but cannot explain the expeditious onset time (in a few minutes to a few hours); much quicker than cellular growth in the pituitary gland. Guerra et al. assumed that PA induced by GnRHa goes through two mechanisms: acute phase and subacute process. Subacute process is induced by multiple factors mentioned above, and the acute phase is initiated by releasing ample hormones from granules in a short time. This would raise the metabolic demands and aggravate local perfusion, resulting in ischemic change and necrosis in adenoma filled with abnormal neovascularization. Pathologic findings manifesting ischemia and necrosis are only confined within adenoma, which is compatible with this hypothesis. Unfortunately, this meticulous dual speculation is impractical to be confirmed by pre- and post-apoplexy brain MRI, which makes room for further investigation [10].

The principle initial acute symptoms are similar to PA owing to other causes (Table 1). Most cases manifested moderate to severe headache, possibly attributed to irritation or stretching of dura mater in the sella supplied by branches of the trigeminal nerve. Other associated clinical features such as nausea and vomiting are also commonly seen and can be extremely severe, resulting in fatal consequences. The present case exhibited upper GI bleeding due to E-C junction tearing, which might be related to severe vomiting. Visual symptoms occur after headache in every patient, including ptosis, ophthalmoplegia, decreased visual acuity, loss of visual field, and anisocoria, consistent with the imaging finding of extrasellar extension.

**Table 1:** Clinical characteristics of patients diagnosed with pituitary apoplexy induced by gonadotropin-releasing hormone agonists

**Authors**	**Patient age (years)**	**Drug**	**Time of onset**	**Pathologic finding**	**Symptoms and signs**	**Treatment**
2013	77	1st Leuprorelin	Hours	Degenerative cells	Severe H/A, N/V, ptosis	Tumor removal (surgery)
Current	Taiwan	3.75 mg		Necrotic debris	Partial ophthalmoplegia	
2010	60	1st Leuprolide	Hours	Necrotic tissue	Mild H/A, blurry vision	Tumor removal (surgery)
Guerra				Hemorrhage;	48 h: ptosis/EOM limitation	
				Stain: LH(+)	9 days: persistent H/A, ptosis, complete ophthalmoplegia	
2007	60	1st Leuprolide	4 h	Pituitary adenoma	H/A, N/V, △MS, and VD, ptosis, mild palsy of CN III.	Medical stabilization
Hands		22.5 mg		Stain: LH(+), FSH(-),	2 weeks: H/A↑,diplopia, weakness	Tumor removal (surgery)
				PRL(-)	3 weeks (untreated): confused, Left CN III, IV, VI paralysis,	
2006	70	1st Leuprolide	10 days	Pituitary adenoma	VD, diplopia and intracranial HTN, ptosis	SMA (little effect)
Massoud,		11.25 mg				3 months: tumor removal (surgery)
2006	61	1st Leuprolide	Few hours	Pituitary adenoma	Severe H/A, N/V, ptosis, diplopia	Tumor removal (surgery)
Davis		30 mg			2 days: ptosis, diplopia, CN III palsy, VD	
2006	68	1st Goserelin,	4-6 h	Pituitary adenoma	Mild H/A,	Tumor removal (surgery)
Blaut		3,6 mg			5-7 days: severe H/A, N/V, △consciousness, diplopia, ptosis,	
2003	69	1st Leuprolide	Hours	Pituitary adenoma	H/A, VD, few days later: DI	Tumor removal (surgery)
Hernandez				Stain: FSH(+)		
2001	67	1st Goserelin	4 h	Pituitary adenoma	H/A, N/V	IV hydrocortisone
Eaton		3.6 mg		Hemorrhage, Necrosis	13 h: Visual loss and severe H/A, mildly confused, HTN,	36 h: Discontinue of Goserelin
				Stain: LH(+), FSH(+)		6 days: cortisone replacement
1997	62	1st Leuprorelin	Soon after injection	Stain: LH(+), FSH(+)	Sudden intracranial HTN	Surgery
Reznik						
1996	74	1st Leuprolide	15 min	Pituitary adenoma	Severe H/A, generalized weakness, N/V.	Steroid therapy
Morsi		7.5 mg		Stain: FSH(+), LH(+)	2 days: consciousness disturbance, ophthalmoplegia	3rd day: decompression of the mass
1995	78	Triptorelin	Few mins	No biopsy	H/A	Discontinue of the GnRH agonist
Chanson		3.75 mg		(CT: suprasellar mass)	24 h: dizziness, partial ophthalmoplegia	Conservative management
1995	83	Goserelin	9 days	No biopsy	H/A, N/V	Replacement therapy: 30 mg prednisolone QD
Ando		3.6 mg		(CT: suprasellar mass)	9-13 days: △ consciousness, hyponatremia, diplopia,	

*CN*, cranial nerve; *CT*, computed tomography; *EOM*, extraocular muscle; *FSH*, follicle-stimulating hormone; *H/A*, headache; *HTN*, hypertension; *LH*, luteinizing hormone; *MRI*, magnetic resonance imaging; *N/A*, not available; *N/V*, nausea/vomiting; *PRL*, prolactin; *RD*, retinal detachment; *RT*, radiotherapy; *SAH*, subarachnoid hemorrhage; *SMA*, somatostatin-like analog; *VD*, visual disturbances; *△*, change.

Moreover, hormonal deficiency is indistinctly detected from physical examination at the time of onset; however, several cases presented with hypopituitarism without adequate hormonal supplement [2,8]. Previous studies showed that hyponatremia occurred in 44% of PA cases, as well as the present case [11]. Randeva et al. postulated that hyponatremia might correlate with hypocortisolism, hypothyroidism, inappropriate secretion of antidiuretic hormone, or a combination of the above [12].

Most pituitary lesions are detected by MRI instead of CT scan (Table 1). Only 29% of cases could be accurately diagnosed by CT scan, whereas the detection rate increases to 57% when combined with MRI. In the present case, brain CT scan could not detect any brain lesion while only MRI confirmed pituitary infarction. A previous study demonstrated MRI is a much more accurate and sensitive tool to identify pituitary adenoma and PA than CT (88% and 21%, respectively) [12]. With regard to MRI, the detection rate can be 100% [12].

Prompt and proper intervention is crucial to these PA patients, especially to those who had consciousness change or cranial nerve palsy. Six patients underwent surgery after several days of initial presentation, four clients received medical treatment at first then changed to surgery because of poor responses, while the first two reported cases had conservative medical management only [2-5,7-10,13]. The outcomes of PA are variable, as according to the previous reports, one expired due to cardiac arrest, another suffered from disseminated malignant melanoma, and one had persistent complications [4,5,8]. Randeva et al. showed that visual acuity could be remarkably improved by transsphenoidal surgery. In patients operated on within 8 days, recovery was complete, but the rate dropped to only 46% after this cut-out point. Fortunately, the present case completely recovered though necessarily receiving transsphenoidal surgery after 10 days of PA attack.

Whether to have every prostate cancer patient have a brain MRI before GnRHa injection is still debatable [4,7,10,11], and is impractical clinically. Intriguingly, a patient underwent adenoma excision while continuously receiving GnRHa injection without the occurrence of PA [9]. This is compatible with the pathological change within the pituitary adenoma, and augments the possibility that PA could be induced by GnRHa in case of pituitary adenoma. The occurrence of PA has been reported in those receiving Leuprorelin, Leuprolide, Triptorelin, and Goserelin injections [2-5,8-10,14]. Nearly every kind of GnRHa has the potential to cause ischemia or bleeding in the pituitary gland.

GnRH analogue is an effective therapy to control PCa. Rarely, it might induce PA in those who have insidious pituitary adenoma coincidentally. Sudden onset headache, nausea, vomiting, and ensuing visual impairment are major characteristics that need to be recognized. When a patient presents these entities after GnRH analogue injection, MRI is the golden tool recommended. Hormonal survey must be done at admission and discharge, in spite of vital signs being stable. In case of visual problems, cranial nerve involvement, or decreased consciousness occurring, the preferred choice is transsphenoidal surgery as early as possible. Hormonal replacement might be prescribed for transient or lifelong use.

## Consent

A written informed consent was obtained from the patient for publication of this case report and any accompanying images.

## Abbreviations

ADT: Androgen deprivation therapy; CT: Computed tomography; EGD: Esophagogastroduodenoscopy; ER: Emergency room; GnRHa: Gonadotropin-releasing hormone agonist; MRI: Magnetic resonance imaging; PA: Pituitary apoplexy; PCa: Prostate cancer; PSA: Prostate specific antigen.

## Competing interests

The authors declare that they have no financial or non-financial competing interests.
